# Top predators negate the effect of mesopredators on prey physiology

**DOI:** 10.1111/1365-2656.12523

**Published:** 2016-04-25

**Authors:** Maria M. Palacios, Shaun S. Killen, Lauren E. Nadler, James R. White, Mark I. McCormick

**Affiliations:** ^1^ ARC Centre of Excellence for Coral Reef Studies and College of Marine & Environmental SciencesJames Cook UniversityTownsvilleQld 4811Australia; ^2^ Institute of Biodiversity, Animal Health and Comparative MedicineCollege of Medical, Veterinary and Life SciencesUniversity of GlasgowGraham Kerr BuildingGlasgowG12 8QQUK

**Keywords:** coral reef fish, metabolic rate, non‐consumptive effects, predator–prey interactions, respirometry, trait‐mediated indirect effects

## Abstract

1. Predation theory and empirical evidence suggest that top predators benefit the survival of resource prey through the suppression of mesopredators. However, whether such behavioural suppression can also affect the physiology of resource prey has yet to be examined.
2. Using a three‐tier reef fish food web and intermittent‐flow respirometry, our study examined changes in the metabolic rate of resource prey exposed to combinations of mesopredator and top predator cues.
3. Under experimental conditions, the mesopredator (dottyback, *Pseudochromis fuscus*) continuously foraged and attacked resource prey (juveniles of the damselfish *Pomacentrus amboinensis*) triggering an increase in prey O_2_ uptake by 38 ± 12·9% (mean ± SE). The visual stimulus of a top predator (coral trout, *Plectropomus leopardus*) restricted the foraging activity of the mesopredator, indirectly allowing resource prey to minimize stress and maintain routine O_2_ uptake. Although not as strong as the effect of the top predator, the sight of a large non‐predator species (thicklip wrasse, *Hemigymnus melapterus*) also reduced the impact of the mesopredator on prey metabolic rate.
4. We conclude that lower trophic‐level species can benefit physiologically from the presence of top predators through the behavioural suppression that top predators impose on mesopredators. By minimizing the energy spent on mesopredator avoidance and the associated stress response to mesopredator attacks, prey may be able to invest more energy in foraging and growth, highlighting the importance of the indirect, non‐consumptive effects of top predators in marine food webs.

## Introduction

Top predators can drive food web dynamics through a variety of direct and indirect effects (Abrams 1995). The indirect effects of predation can be observed in three‐tier trophic cascades, where a direct negative link between the top predators and intermediate‐level species (e.g. mesopredators, mesoconsumers) often indirectly favours the next consecutive trophic level of resource prey (Werner & Peacor 2003; Schmitz, Krivan & Ovadia 2004). For instance, correlative evidence from temperate forests suggests wolves limit habitat use and grazing patterns of ungulate herbivores, which in turn indirectly enhances the survival and recruitment of native vegetation (Ripple *et al*. 2001). Few studies have assessed a fully trait‐mediated pathway in which successive predator–prey interactions are driven by predation risk, with impacts on the behavioural, physiological or morphological traits of the species. Recently, Gordon *et al*. (2015) and Palacios, Warren & McCormick (2016) showed that in particular desert and coral reef food webs, top predators can alter the non‐consumptive effects (predation risk) of mesopredators and indirectly affect the behaviour of resource prey (e.g. increased habitat breadth, reduced anti‐predator behaviour). Given that organisms make costly energetic trade‐offs between predator avoidance and self‐maintenance activities (reviewed by Lima 1998; Brown & Kotler 2004), top predators could have positive indirect effects on the lifetime fitness of resource prey (e.g. mating success, fecundity, reproductive rate). However, this can only be determined by detailed examinations of the effects of risk‐induced trophic cascades on different behavioural, physiological and morphological traits of prey.

Prey physiology is strongly affected by the presence of predators (reviewed by Hawlena & Schmitz 2010; Zanette, Clinchy & Suraci 2014). In vertebrates, physiological responses to predation risk include altered cardiovascular activity, ventilation and metabolism (e.g. Ward *et al*. 1996; Cooke *et al*. 2003; Hawkins, Armstrong & Magurran 2004; Steiner & Van Buskirk 2009). These physiological mechanisms can improve the prey's probability of escaping an attack, but can be energetically costly and may decrease the surplus of energy available for other tasks such as activity, growth, maintenance or reproduction (Houston, McNamara & Hutchinson 1993; DuRant, Hopkins & Talent 2007). Consequently, chronic and/or frequent exposure to predation stress can reduce the energy allocation for essential physiological functions (Hawlena & Schmitz 2010). For example, under chronic predation risk, snowshoe hares experience a reduction in their body condition index, leucocyte counts and reproductive output (Boonstra *et al*. 1998; Sheriff, Krebs & Boonstra 2009). In addition, larval and juvenile marine fishes that experience frequent exposure to predator cues display reduced growth and lipid stores (Killen & Brown 2006; Killen, Gamperl & Brown 2007). While non‐consumptive predator–prey interactions are physiologically costly for prey, it is yet unknown whether such costs can be ameliorated when the predator itself is under behavioural suppression by a higher‐level predator. Given that the anti‐predator response of animals is proportional to the level of predation risk (Helfman 1989), we hypothesize that any restrictions in the activity and foraging of the mesopredator should reduce predator‐induced stress and energy expenditure of the prey.

To address this knowledge gap, we explored a potential mechanism through which risk‐driven effects may cascade to influence the metabolic rate of resource prey. Using a three‐level food web of coral reef fishes as a model system, we experimentally examined how risk elicited by a top predator altered mesopredator behaviour and consequently modified their influence on resource prey activity and oxygen uptake. We specifically aimed to (i) determine whether acute predation risk by a top predator (coral trout) affects the behaviour of a mesopredator (dottybacks) and (ii) quantify how the altered behaviour of the mesopredator affects the metabolic rate (estimated oxygen uptake) of resource prey (damselfish juveniles). The terms ‘top predator > mesopredator > resource prey’ refer to the hierarchy and trophic status among the three species used in the study and are not meant to imply that they have a fixed trophic category in their natural ecosystem (e.g. the coral trout could be the top predator in one system but the mesopredator in another). At any given point, these terms could be replaced by ‘high trophic‐level species > intermediate‐level species > bottom‐level species’, respectively.

## Materials and methods

### Experimental Overview

Changes in the metabolic rate (oxygen uptake) of damselfish juveniles were measured and compared among six experimental treatments crossing the presence of a mesopredator (2 levels: dottyback, goby) with a top predator (3 levels: coral trout, thicklip wrasse, empty tank). The goby and thicklip wrasse served as non‐predator species to control for the meso‐ and top predator, respectively. Behavioural observations were recorded both on the damselfish juveniles and on the mesopredators (dottybacks/gobies). Eight to nine replicate trials were undertaken for each treatment, with all fish being tested only once to maintain independence among trials. Routine metabolic rate was calculated given its common use as an indicator of stress and energy expenditure in response to predation risk (Chabot, Gagnon & Dixon 1996; Ward *et al*. 1996; Holopainen *et al*. 1997; Steiner & Van Buskirk 2009) and their correlation to a number of ecologically relevant behaviours and life‐history traits (Biro & Stamps 2010; Burton *et al*. 2011; Killen *et al*. 2013).

### Study Species and Fish Handling

Juveniles of the common Indo‐Pacific damselfish, *Pomacentrus amboinensis,* were used as the resource prey. This benthic species is a site‐attached omnivorous demersal spawner with a bipartite life history. When the larvae (10–15 mm SL; Kerrigan 1996) settle to shallow reefs during the austral summer months (October–January), they are subject to extremely high rates of predation by small reef piscivores such as cods, dottybacks and lizardfishes (Almany & Webster 2006). These damselfish juveniles can learn to recognize reef predators, have strong anti‐predator behaviour and exhibit threat‐sensitive responses to predation risk (Holmes & McCormick 2011). The dottyback (*Pseudochromis fuscus*) was used as the focal mesopredator species, as it is a small (10 cm TL) site‐attached carnivore that voraciously consumes newly settled fishes using ambush and pursuit techniques (Feeney *et al*. 2012). It acclimates well to aquarium conditions and is known to respond to visual and chemical cues from top predators (Palacios, Warren & McCormick 2016). The leopard coral trout (*Plectropomus leopardus*) was used as the top predator species. This large (>30 cm SL) reef piscivore is relatively common on the Great Barrier Reef (GBR; Ayling, Samoilys & Ryan 2000) and consumes predominantly small‐sized reef fish (3–7 cm SL; St. John 2001). The non‐piscivorous reef fish species selected to experimentally control for the presence of the meso‐ and top predator were the white‐barred goby (*Amblygobius phalaena*) and the thicklip wrasse (*Hemigymnus melapterus*), respectively. The goby (<15 cm TL) feeds mainly on algae and copepods (Sano 1984), while the wrasse (>30 cm TL) usually consumes small crustaceans, polychaete worms and molluscs (Randall 2013). Although both non‐predators are frequently found around patch reefs and in close proximity to newly settled fish, they are not known to prey on them.

All fishes were collected from the lagoon of Lizard Island (14°40′S, 145°28′E), northern GBR, during the second week of November 2014. Damselfish juveniles (13·7 ± 0·08 mm, mean SL ± SE, *N* = 54) were captured from the reef edge with light traps moored overnight, while both dottybacks (7·03 ± 0·05 cm, mean TL ± SE, *N* = 26) and gobies (7·5 ± 0·1 cm, mean TL ± SE, *N* = 28) were collected from patch reefs by SCUBA divers using hand nets and a mild anaesthetic clove oil solution. Specimens of *P. leopardus* (39·4 ± 1·07 cm, mean TL ± SE, *N* = 5) and *H. melapterus* (28·3 ± 2·3 cm, mean TL ± SE, *N* = 4) were caught using hand lines (with barbless hooks) and barrier nets, respectively. Fishes were maintained at the Lizard Island Research Station in separate holding tanks. Coral trouts and thicklip wrasses were individually kept in 300‐L round tanks, dottybacks were isolated individually in porous 1‐L containers in groups of 10 in 68‐L tanks, and all of the damselfish juveniles were kept together in a 22‐L aquarium (~3 fish per L). All tanks had a flow‐through seawater system at ambient temperatures (27·5–29 °C) and light photoperiods (12 h light: 12 h dark). Damselfish juveniles were fed *Artemia* spp. twice daily, while the rest of the fishes were fed prawn or squid.

Before the onset of experimental procedures, damselfish juveniles were trained to recognize cues from the dottybacks as their collection prior to reef settlement may have prevented them from learning the identity of reef‐associated predators. Naïve juvenile fishes can learn the identity of a novel predator by simultaneously presenting conspecific damage‐released chemical cues (indicative of threat) with visual and/or chemical cues of a predator (Brown & Chivers 2005). Similar to the protocols followed by McCormick & Holmes (2006) and Lönnstedt *et al*. (2012), damselfish juveniles were trained by exposing them concurrently to a variety of cues, including 10 mL of the conspecific damage‐released chemical cues, 30 mL of the dottyback odour and a live dottyback placed in a sealed ziploc bag (serving as a visual cue). After 10 min, all cues were removed from the tank and water flow was restored. To prepare the damage‐released chemical cues, three damselfish per training session (12–14 mm SL) were euthanized with a quick blow to the head and placed in a Petri dish where 10 superficial cuts were made to the skin of each donor fish (5 cuts per flank). Fish were then rinsed with 10 mL of seawater (previously obtained from their tank) creating a solution of damage‐released alarm cues. To obtain the mesopredator odour, four dottybacks were randomly selected and kept for at least 12 h in a tank containing 4 L of aerated seawater.

### Experimental Set‐Up

**Figure 1:**
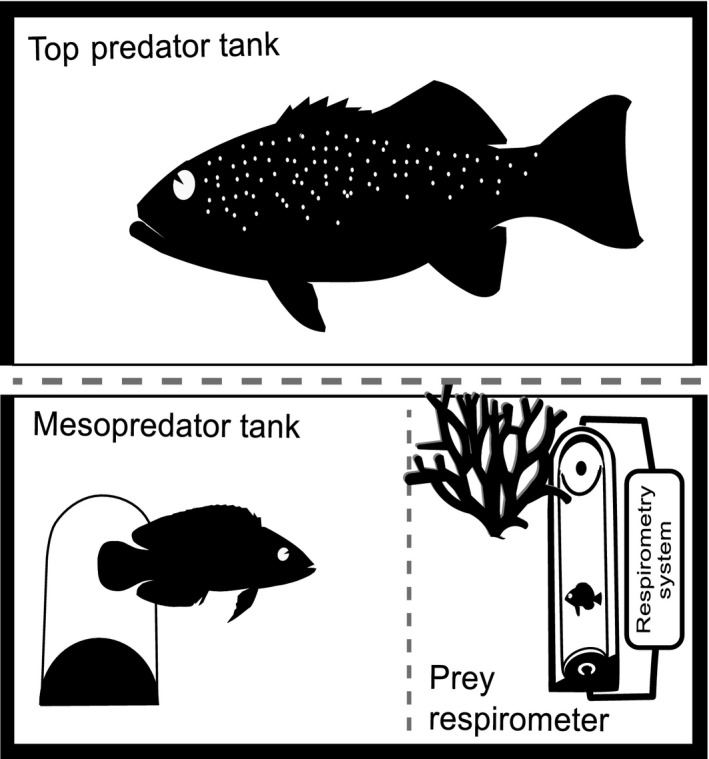
Experimental set‐up used to assess the indirect interactions between a three‐level food web of coral reef fish. The set‐up includes (a) a top predator tank to hold the top predator (coral trout, *Plectropomus leopardus*) or the non‐predator (thicklip wrasse, *Hemigymnus melapterus*) and (b) a mesopredator tank where the mesopredator (dottyback, *Pseudochromis fuscus*) or the small non‐predator (goby, *Amblygobius phalaena*) could swim freely and interact with top predator and/or prey. Each mesopredator tank contained a layer of sand, a shelter for the mesopredator, a resin branching coral and a respirometry chamber to hold the resource prey (damselfish juveniles, *Pomacentrus amboinensis*). A removable opaque panel was positioned between the top predator and mesopredator tanks (grey discontinuous line) allowing an exchange of visual cues only when it was removed. A second panel divided the mesopredator tank, and only with its removal could the mesopredator approach and interact with the damselfish.

Experiments were undertaken in four pairs of replicate glass tanks (25 × 60 cm; 30 cm water). Each pair of tanks consisted of a mesopredator tank and a top predator tank positioned next to each other along their longest side (Fig. 1). Except on the face they shared, both tanks were completely shielded from external disturbances by opaque curtains. Each mesopredator tank contained a layer of sand, a shelter for the mesopredator (PVC tube: 8 cm length × 3 cm diameter), a resin branching coral (14 × 11·5 × 5 cm; item no. 21505; Wardleys/TFH) and a sealed glass respirometry chamber for the damselfish juvenile (described below). Two removable opaque panels were used to modulate the interactions and cue exchange between the fish. The first panel was positioned between the mesopredator and the top predator tanks allowing an exchange of visual cues only when it was removed. A previous study with the same study species showed that visual cues from the top predator are sufficient to achieve behavioural suppression of the dottyback (Palacios, Warren & McCormick 2016). The second panel divided the mesopredator tank transversally into two sections, separating the mesopredator and its shelter from the damselfish juvenile. Only when this panel was removed, could the mesopredator approach and interact with the damselfish juvenile. Video cameras installed over each pair of experimental tanks recorded the behaviour of each fish. As in the holding tanks, all experimental tanks had constant flow‐through seawater at ambient temperature.

Intermittent‐flow respirometry was used to measure oxygen uptake of damselfish juveniles as proxy for aerobic metabolism. This technique allows continuous monitoring of dissolved oxygen levels inside a respirometry chamber that is intermittently flushed with oxygenated water to measure oxygen decline in the absence of hypoxia (Svendsen, Bushnell & Steffensen 2016). Oxygen uptake is a good approximation for aerobic metabolic rate as oxygen is consumed in the breakdown of stored energy in order to fuel many of the most important processes that affect fitness, including locomotor activity, growth and maintenance (Chabot, Steffensen & Farrell 2016; Nelson 2016). In this study, respirometers consisted of individual cylindrical glass chambers (11 cm length × 2 cm diameter; total volume of chamber plus associated tubing = ~ 30mL) protected externally with half‐cylinders of clear acrylic (11 cm length × 5·5 cm radius). Water flow through the chambers was driven by an external pump set to alternately turn on (2 min) and off (8 min) throughout the measurement periods. This allowed water oxygen content to be measured every 2 s for 8 min while the respirometer was in the closed state, after which the respirometer was flushed with aerated water for 2 min to prevent it from reaching hypoxic levels. Water mixing within each respirometer was achieved with a pump that moved water through the chamber and around an external circuit of gas‐impermeable tubing. Also located within the circuit for each respirometer was a flow‐through cell that housed an oxygen‐sensing optode attached to an oxygen sensor (Firesting 4‐Channel oxygen metres; Pyro‐Science, Aachen, Germany) and a computer. To correct for background bacterial respiration, oxygen uptake was recorded for 30 min at the beginning and end of each trial in each chamber without fish. Every day the respirometers, flow‐through cells and tubing were thoroughly cleansed with soap, bleach and hot water.

### Experimental Protocol

All juveniles were starved for 24 h prior to experimentation in order to ensure that they were in a post‐absorptive state (Niimi & Beamish 1974). Experimental trials began with a pre‐stimulus period, in which a damselfish juvenile was introduced into the respirometer and left undisturbed for 2 h while recording its oxygen uptake and activity. After 2 h, the assigned mesopredator (dottyback/goby) and top predator (coral trout/ thicklip wrasse) were introduced into the tanks and left to acclimate for 20 min. The post‐stimulus period was initiated by removing the two opaque panels, thereby allowing the mesopredator to simultaneously (i) interact with the damselfish juvenile and (ii) receive visual cues from the top predator. For the following 2 h, the behaviour of the mesopredator and the post‐stimulus oxygen uptake and activity of the damselfish were recorded. Each trial lasted approximately 4 h 20 min. All damselfish juveniles were then weighed to determine wet body mass. A total of 53 trials were executed over 9 days, running simultaneously four trials in the morning and four in the afternoon. Every day the six experimental treatments were randomly assigned to the two periods of the day (am/pm) and the four pairs of replicate tanks.

### Metabolic and Behavioural Assessment

Measures of metabolic rate (oxygen uptake; mg O_2_ h^−1^) and activity (line crosses) were estimated for each damselfish juvenile. Routine metabolic rates were estimated as the mean level of oxygen uptake in the 1 h before and after exposure to the predator cues. Rates during each closed phase were calculated using linear least‐squares regression, excluding the first and last minute of each closed phase. In total, six measures of oxygen uptake were collected per hour and used to calculate the oxygen uptake of each prey damselfish. Activity was measured by quantifying the number of times the damselfish juvenile crossed five equidistant lines that transversally divided the respirometer chamber into six 1·8‐cm‐width sections. Line crosses were only assessed during the first 10 min of the pre‐ and post‐stimulus 1‐h periods selected for metabolic analysis. Pilot observations showed this sample period (10 min) was representative of the activity of the damselfish during the correspondent hour.

Three behavioural attributes of the mesopredators (dottybacks/gobies) were quantified from the 10‐min post‐stimulus period: (i) time spent inside shelter (min), (ii) time spent near the respirometer containing the prey (min) and (iii) number of strikes to the respirometer. The time near the respirometer included all of the time the mesopredator was closer than one body length from the chamber, while the number of strikes considered all of the attacks in which the mesopredator hits the chamber with its mouth.

### Statistical Analysis

Changes in the damselfish metabolic rate between the pre‐ and post‐stimulus observation periods were calculated (Δ O_2_ uptake; mg h^−1^) and compared using a two‐factor analysis of covariance (ancova) with mesopredators (2 levels: dottyback, goby) and top predators (3 levels: coral trout, thicklip wrasse, empty tank) as factors. Damselfish wet body mass (g) and change in activity (Δ line crosses) were used as covariates in the analysis to correct for the effects of body size and movement. Additionally, a general linear model (GLM) was used to examine the relationship between the change in activity (Δ line crosses) and the change in oxygen uptake (Δ O_2_ uptake; mg h^−1^) of damselfish juveniles exposed to the six treatments. Mesopredator behaviour was analysed among treatments using a two‐factor analysis of variance (anova) with either time spent inside shelter or time spent near the respirometer as dependent variables. Gobies never attacked the damselfish juveniles, so the number of strikes was analysed only for the dottybacks through a one‐way anova. All significant differences detected were further explored using Tukey's HSD post hoc test for unequal N. Residual analyses were used to examine whether the data satisfied the assumptions of normality and homoscedasticity. Data from the damselfish juveniles were normal and homoscedastic; however, data from the mesopredators were square‐root‐transformed to meet the assumptions of parametric tests.

## Results

**Figure 2:**
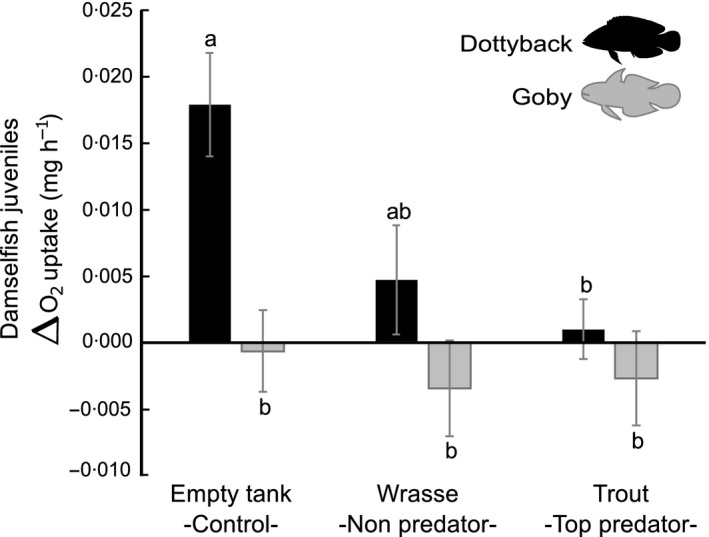
Change (mean ± SE) in the oxygen uptake (ΔMO_2_; mg O_2_ h^−1^) of damselfish juveniles between 1 h pre‐ and post‐stimulus periods. During the post‐stimulus periods, damselfish were exposed to the combination of a mesopredator (dottyback/goby) and a top predator (coral trout/thicklip wrasse/empty tank). The goby and thicklip wrasse served as non‐predator species to control for the meso‐ and top predator, respectively. Black bars correspond to the dottybacks (mesopredator), while grey bars to the gobies (small non‐predator). A positive value indicates an increase in oxygen uptake, while a negative value indicates a decrease. Bars with the same lowercase letter did not differ significantly according to the Tukey's HSD *post hoc* test.

Changes in the oxygen uptake of the damselfish juveniles (ΔMO_2_) between the pre‐ and post‐stimulus periods were significantly influenced by the mesopredator (dottyback/goby), the top predator (coral trout/thicklip wrasse/empty tank) and their interaction (Fig. 2, Table 1a; ancova main effects and interaction, *P* < 0·05). When damselfish juveniles were exposed to the goby (small non‐predator), their oxygen uptake remained relatively constant independent of the presence of an empty tank, a wrasse or a trout (Fig. 2; grey bars are not significantly different, Tukey's HSD test, *P* > 0·05). Nevertheless, in the presence of a dottyback (mesopredator), the oxygen uptakes of damselfish were influenced by the top predator treatments (Fig. 2; black bars significantly different, Tukey's HSD test, *P* < 0·05). Oxygen uptakes of damselfish increased by 38 ± 12·9% (mean ± SE) if they were exposed to the dottyback alone (cues of an empty tank), yet remained constant if the dottyback was under the effect of a coral trout (cues of a top predator). Damselfish increased oxygen uptake to intermediate levels if the dottyback was under the effect of the wrasse (Fig. 2).

**Table 1:** (a) Parameter estimates of the ancova used to test the change in oxygen uptake (ΔMO_2_; mg h^−1^) of the damselfish juveniles (resource prey) exposed to six treatments combining a mesopredator (dottyback/goby) with a top predator (coral trout/thicklip wrasse/empty tank). The goby and thicklip wrasse served as non‐predator species to control for the meso‐ and top predator, respectively. The variables body mass and change in activity were used as covariates in the assessment of metabolic change for the damselfish. (b) Parameter estimates are also shown for the GLM examining the relation between the change in activity (ΔAc; line crosses) and the change in oxygen uptake (ΔMO_2_; mg h^−1^) of damselfish juveniles exposed to the six treatments

Sources of variation	SS	DF	MS	*F*	*P*
a. ancova					
(a) Mesopredator	1·68E‐03	1	1·68E‐03	19·43	0·000***
(b) Top predator	7·45E‐04	2	3·72E‐04	4·30	0·020*
(a) × (b)	5·90E‐04	2	2·95E‐04	3·41	0·042*
Covariates					
Body mass (g)	1·19E‐04	1	1·19E‐04	1·37	0·248ns
Δ activity (line crosses)	4·20E‐04	1	4·20E‐04	4·85	0·033*
Error	3·64E‐03	42	8·66E‐05		
b. GLM					
(a) Treatment	3·03E‐03	5	6·05E‐04	6·52	0·000***
(b) Δ activity (line crosses)	2·52E‐04	1	2·52E‐04	2·71	0·108ns
(a) × (b)	2·25E‐04	5	4·49E‐05	0·48	0·786ns
Error	3·53E‐03	38	9·29E‐05		

Asterisks indicate significant differences where *P ˂ 0·05, **P ˂ 0·01 and ***P ˂ 0·001.

**Figure 3:**
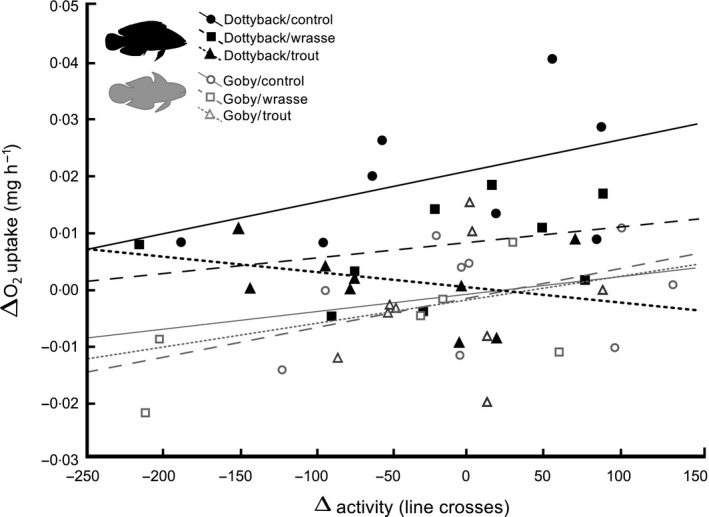
Relationship between the change in activity (ΔAc; line crosses) and the change in oxygen uptake (ΔMO_2_; mg h^−1^) of damselfish juveniles exposed to six treatments combining the presence of a mesopredator (dottyback/goby) and a top predator (coral trout/thicklip wrasse/empty tank) during the post‐stimulus period. The goby and thicklip wrasse served as non‐predator species to control for the meso‐ and top predator, respectively. Black symbols correspond to the dottybacks (mesopredator), while grey symbols to the gobies (small non‐predator). For both axes, positive values indicate an increase in the variable (activity or O_2_ uptake) during the post‐stimulus period, while negative values indicate a decrease.

Changes in activity of the damselfish (ΔAc; line crosses) did have a significant effect on the ΔMO_2_ (Table 1a; *F*_1,42_ = 4·85, *P* < 0·05). Overall, the ΔAc were positively correlated with the ΔMO_2_ (Fig. 3). Although the relation between the ΔAc and the ΔMO_2_ did not statistically differ among the six treatments (Table 1b; GLM interaction, *F*_5,38_ = 0·48, *P* > 0·05), there were interesting qualitative differences in the responses observed during exposure to the goby and the dottyback (Fig. 3). In the presence of the non‐predatory goby, decreases in the activity (negative change) of the damselfish were most often associated with decreases in oxygen uptake. However, in the presence of the mesopredator (dottyback), many damselfish increased oxygen uptake despite reducing their activity. Further, damselfish that increased their activity usually had higher levels of oxygen uptake if exposed to a dottyback alone (control) or a dottyback under the effect of the wrasse (Fig. 3).

**Figure 4:**
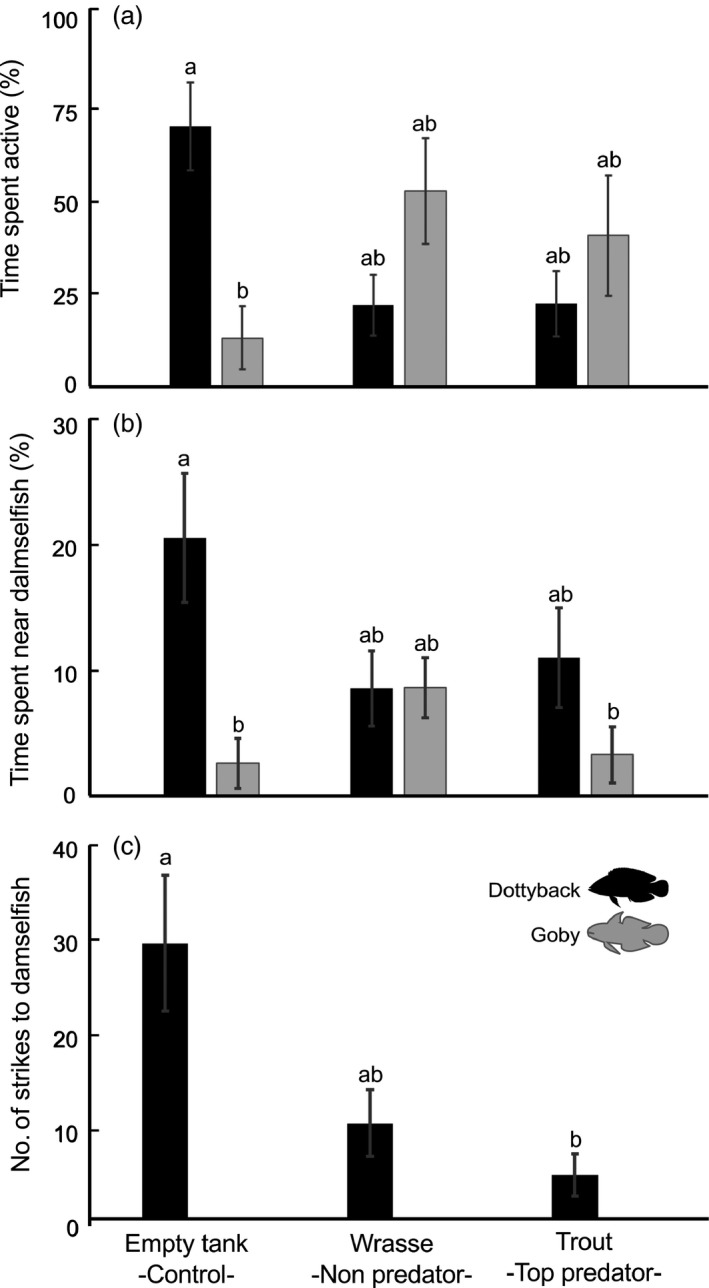
Mean (± SE) (a) time spent active (%), (b) time spent near the damselfish chamber (%) and (c) number of strikes (#/10 min) recorded for dottybacks (mesopredator) and gobies (small non‐predator) exposed to an empty tank (control conditions), a thicklip wrasse (large non‐predator) or a coral trout (top predator). Black bars correspond to dottybacks, while grey bars to gobies. Bars with the same lowercase letter did not differ significantly according to the Tukey's HSD *post hoc* test.

The behaviour of the dottyback and goby was affected differently by the top predator (Table 2; anova interaction, *P* < 0·05, Fig. 4a,b). Under control conditions (cues of an empty tank), dottybacks were significantly more active than the gobies, spending more than 70% of the time exploring the arena and constantly approaching the damselfish chamber (~20% of the time, Fig. 4a,b; black and grey bars in the control treatment are significantly different, Tukey's HSD test, *P* < 0·05). However, under the effect of a large fish (either wrasse or trout), the behavioural differences disappeared, as dottybacks and gobies spent a similar percentage of time active in the tank and near the damselfish (Fig. 4a,b). Gobies never attacked the damselfish (as expected by their non‐piscivorous food preferences), so the number of strikes was only recorded for the dottybacks. Under control conditions, dottybacks frequently struck at the damselfish chamber. However, when dottybacks were simultaneously exposed to the trout, the total number of strikes was significantly reduced by 83·6% (Fig. 4c; Table 2; anova,* F*_2,22_ = 6·9, *P* < 0·01). Dottybacks struck at the damselfish an intermediate number of times when exposed to the wrasse (top predator control).

**Table 2:** Parameter estimates of the anovas examining three behavioural attributes (time spent active, time spent near damselfish, No. of strikes) of the mesopredators (dottybacks/gobies) exposed to a top predator (coral trout/thicklip wrasse/empty tank). The thicklip wrasse served as non‐predator species to control for the top predator

Time spent active (s)	SS	DF	MS	*F*	*P*
(a) Mesopredator	367·61	1	367·61	0·07	0·793ns
(b) Top predator	3541·58	2	1770·79	0·34	0·717ns
(a) × (b)	80946·19	2	40473·10	7·67	0·001**
Error	248131·54	47	5279·39		

Time spent near damselfish (s)	SS	DF	MS	*F*	*P*
(a) Mesopredator	71·34	1	71·34	10·12	0·003**
(b) Top predator	11·78	2	5·89	0·84	0·440ns
(a) × (b)	53·78	2	26·89	3·81	0·029*
Error	345·41	49	7·05		

No. of strikes to damselfish	SS	DF	MS	*F*	*P*
Top predator	3·40	2	1·70	6·90	0·005**
Error	5·42	22	0·25		

Asterisks indicate significant differences where *P ˂ 0·05, **P ˂ 0·01 and ***P ˂ 0·001.

## Discussion

Our study demonstrated a cascade of trait‐mediated effects through which top predators indirectly negated the effect of mesopredators on prey metabolic rate. Under experimental conditions, the mesopredator (dottyback) frequently attacked resource prey (damselfish juveniles) triggering a marked increase in their metabolic rate. As hypothesized, however, acute risk from the top predator (visual cues of a coral trout) restricted the mesopredator behaviour (reduction in activity and feeding strikes), indirectly allowing resource prey to reduce physiological stress and minimize routine metabolic rate.

The cascade of non‐consumptive effects documented here begins with the behavioural suppression of the mesopredators by the top predator. Dottybacks exposed to acute predation risk from the top predator allocated less time to foraging (i.e. less time near the prey and a lower number of attacks) as the threat of predation from the coral trout induced them to shelter and remain inactive most of the time. Similar trade‐offs and behavioural changes have been observed in a wide range of taxa, as prey often reduce their activity, space use and foraging under predation risk (e.g. Orrock, Danielson & Brinkerhoff 2004; Valeix *et al*. 2009). Interestingly, when foraging restrictions occur on intermediate‐level species, they often result in a reduction in the consumptive effects they impose on the next lower trophic level (e.g. Schmitz, Beckerman & O'Brien 1997; Turner 1997; Trussell, Ewanchuk & Bertness 2002). For example, in coral reef ecosystems, top predator fishes are known to restrict foraging of mid‐size carnivores (e.g. coneys, *Cephalopholis fulva*; graysbys, *C. cruentata*; Stallings 2008) and mid‐size grazers (e.g. blackbar damselfish, *Plectroglyphidodon dickii*; Madin, Gaines & Warner 2010), thereby having positive indirect effects on the survival of juvenile fishes and density of algae, respectively.

In the present study, the behavioural suppression of the mesopredator by the top predator modified its non‐consumptive impact on the physiology of the resource prey. Active and foraging mesopredators induced an increase in the routine metabolic rate of the resource prey that was likely due to at least two main sources: (i) increased locomotor activity while avoiding the mesopredator strikes and (ii) an increased autonomic stress response. Similar predator‐induced respiratory responses have been recorded for many vertebrates (Chabot, Gagnon & Dixon 1996; Ward *et al*. 1996; Holopainen *et al*. 1997; Hawkins, Armstrong & Magurran 2004). Animals have a finite‐energy budget to distribute between self‐maintenance and investment processes (Stearns 1992; Ricklefs & Wikelski 2002); therefore, increases in routine oxygen uptake may limit the allocation of energy to somatic growth, reproduction and storage (Houston, McNamara & Hutchinson 1993; DuRant, Hopkins & Talent 2007). Negative effects of predation risk could be further exacerbated by other primary and secondary physiological stress responses (i.e. increased cortisol, heart rate, ventilation), which are known to alter food assimilation, body condition and immunocompetence (Höjesjö, Johnsson & Axelsson 1999; Pijanowska & Kloc 2004; Killen & Brown 2006; Killen, Gamperl & Brown 2007; Sheriff, Krebs & Boonstra 2009). These effects may be especially problematic for early juvenile fishes which face a large number of predators (Caley 1993; Almany & Webster 2006; Hixon 2015) and are under pressure to (i) rapidly grow and escape gape‐limited predation (Bailey 1989; Holmes & McCormick 2010), (ii) increase the array of food items they can potentially utilize (Fuiman 1994) and (iii) improve their body condition to better cope with additional stressors (e.g. competition; Booth & Beretta 2004; Hoey & McCormick 2004). Frequent interruptions to routine foraging (behavioural restrictions), with accompanying increases in metabolic rate due to stress and activity, could have consequences for juvenile fitness and survival. Thus, we hypothesize that by minimizing increases in prey metabolic rate caused by mesopredator attacks, top predators could have a positive indirect effects on the physiology and perhaps fitness of resource prey.

Interestingly, the top predator species was not the only treatment to alter the behaviour of the mesopredators and affect the metabolic rate of the prey. Results showed that mesopredators exposed to the invertivorous wrasse reduced their foraging activity, which lead to an intermediate level of oxygen uptake by resource prey. We consider, however, that the behavioural changes of the mesopredators were not triggered by an anti‐predator response (as occurs in the presence of coral trout), but by their engagement in ‘inspection behaviours’ towards the wrasse. Many vertebrates commonly inspect large, novel species (through tentative approaches and follow‐ups) to acquire extra information on the potential threat that they pose (e.g. FitzGibbon 1994; Fishman 1999; Walling *et al*. 2004). In this case, although the invertivorous wrasse did not represent a threat to the mesopredator, it played a key role in ‘distracting’ the mesopredator, limiting its attacks on the resource prey and indirectly allowing prey to mount a lower physiological stress response. These findings are in line with previous studies, which suggest that large‐sized individuals (regardless of their trophic status or diet) could hinder the foraging impact of intermediate‐sized species on resource prey (Marsh‐Hunkin, Gochfeld & Slattery 2013; Palacios, Warren & McCormick 2016). However, further research would be essential to determine the duration of mesopredator ‘inspection behaviours’ of novel species and to what extent it can divert the attention of animals from other activities such as foraging.

Our results show a potential mechanism through which top predators, or even large‐sized non‐predatory individuals, can indirectly influence the physiology of bottom trophic‐level species. However, the nature and magnitude of the indirect effects reported should be considered in the context of the trials (e.g. procedure, experimental set‐up) and characteristics of the species employed. It must be taken into account that anti‐predator behaviours and physiological responses can depend on the intrinsic phenotypic traits of the animal (e.g. size, body condition; Lönnstedt & McCormick 2011; Preisser & Orrock 2012; Wormington & Juliano 2014), the level of predation risk present in the sampled population (high vs. low risk environments; Brown, Gardner & Braithwaite 2005; Bell, Henderson & Huntingford 2010; Clinchy *et al*. 2011) and the duration of the predation risk (acute vs. chronic; Holopainen *et al*. 1997; Steiner & Van Buskirk 2009). Experimental manipulations are an useful initial step in understanding the non‐consumptive links within trophic levels (Schmitz, Krivan & Ovadia 2004); however, future studies should address how these indirect effects may apply to more natural and complex scenarios. Field‐based approaches will be indispensable to accurately extrapolate our results to coral reef systems where (i) food webs have multiple complex trophic levels, (ii) consumptive and non‐consumptive interactions can occur simultaneously and (iii) the duration and strength of predation risk is highly variable. Although much evidence exists on the effects of predators on prey behaviour and physiology (single predator–prey interaction; reviewed by Hawlena & Schmitz 2010; Zanette, Clinchy & Suraci 2014), future research should build on results presented here to determine how these effects may be modified by multiple trophic levels of predator–prey interactions.

In summary, our study examined whether the non‐consumptive effects of top predators could cascade through the food web to impact the physiology of resource prey. We found that acute predation risk from a top predator reduced the predatory behaviour of the mesopredators and thereby minimized the impact of the mesopredator on the oxygen uptake, activity and physiological stress of the resource prey. These results suggest that a release of mid‐ranking piscivores, due to the overexploitation of large piscivores and the alteration of predation risk in marine food webs (Madin *et al*. 2016), could largely increase their non‐consumptive impact on bottom resource prey. Increasing levels of predation risk from mesopredators are expected to reduce feeding opportunities, which along with high self‐maintenance costs could impair the fitness, growth and survival of the recruiting fishes. Although logistically challenging, the incorporation of predator‐induced plasticity into theoretical models (e.g. Abrams 1990; Bolker *et al*. 2003) and empirical research of predator–prey interactions (e.g. Schmitz, Beckerman & O'Brien 1997; Heithaus *et al*. 2007) is critical for the realistic evaluation of the effects of predators on prey populations and community dynamics.

## Data accessibility

Data are publicly available at the Tropical Data Hub Research Data repository. http://dx.doi.org/10.4225/28/56E8E899DD85B (Palacios *et al*. 2016).
